# Emphysema and Interstitial Pneumonia in Rheumatoid Arthritis

**DOI:** 10.1016/j.ebiom.2018.01.040

**Published:** 2018-02-01

**Authors:** Fumikazu Sakai

**Affiliations:** Department of Diagnostic Radiology, International Medical Center, Saitama Medical University, Hidaka, Saitama 350-1298, Japan

Emphysema (cystic change) is well noted in never-smokers and develops following inhalation of organic or nonorganic exogenous noxious substances or as the sequela of destructive change in the airways or fragile lung tissue, for example, vascular type Ehlers-Danlos syndrome. In patients with interstitial pneumonia (IP), the cysts may arise in response to either the inhalation of a noxious substance that causes the IP or as a consequence of the disease itself.

In a recent article in *EBioMedicine*, among patients with comorbid rheumatoid arthritis and interstitial pneumonia (RAIP), Jacob et al. report similar findings of emphysema in high-resolution computed tomography (HRCT) of more than 25% of patients who never smoked and those with RAIP who were smokers (Jacob et al., 2018). In addition, those with RAIP who never smoked also shared the same characteristic features in pulmonary function tests as patients with coexistent interstitial pneumonia and emphysema (combined pulmonary fibrosis and emphysema [CPFE]) who smoked.

Cottin et al. have redefined CPFE as a syndrome consisting of emphysema in the upper lung with varying patterns of interstitial fibrosis (most frequently that of usual interstitial pneumonia [UIP]) in the lower lung that demonstrates characteristic profiles in pulmonary function tests, including findings of relatively preserved forced vital capacity (FVC) and low diffusion capacity (DL_CO_), and may be associated with a poor prognosis (Cottin et al., 2005).

Large cystic changes with distinct but relatively thin wall frequently observed in pathologic specimens of smoker's lung, that have been described as emphysema with fibrosis and air space enlargement with fibrosis (AEF) (Kawabata et al., 2008) and other names, these cysts are sometimes related to the airway on pathological specimens (Kawabata et al., 2008; Inomata et al., 2014). Degree of AEF is significantly related with smoking index (Kawabata et al., 2008). Pathologic manifestations of CPFE vary from the simple coexistence of emphysema and idiopathic interstitial pneumonia (IIP) to IP that is deeply related to smoking. HRCT features of CPFE include large cystic change with relatively thin but distinct wall in upper lung and fibrotic areas of lower lung and one of pathological basis of cystic changes is AEF (Watanabe et al., 2015).

Although Jacob et al. observed similar morphologic features on HRCT, findings in pulmonary function tests, and clinical features (poor prognosis) between patients with RAIP who never smoked and those with CPFE, the similarities did not assure the same morphogenesis of “emphysema” in these patients, and this series offered no confirmation of histopathological basis of emphysematous cysts (Jacob et al., 2018). Nevertheless, HRCT can reflect the pathological process of diffuse lung disease, and similar CT features of cysts between smokers and non-smokers may reflect a common morphogenesis. In never-smokers with RAIP, emphysematous cysts are frequently seen in IP of usual pattern (UIP) and may be seen in IP of other patterns that show a spurious UIP pattern at HRCT as a result of complicated emphysema that mimics honeycombing (Akira et al., 2009).

Jacob et al. demonstrated that emphysema is an independent predictor of survival in never smoker RAIP patients and the findings are in line with the results of Cottin et al. (REF) who described CPFE showed a poor prognosis when compared to IP without emphysema. It therefore appears that interstitial pneumonia with prominent destructive changes may show a poor prognosis regardless of the cause of destruction (Cottin et al., 2005).

Patients with rheumatoid arthritis commonly experience disease of the airways, such as bronchiectasis, bronchiolitis, and interstitial lung disease (Mori et al., 2008) as a result of inflammation that destroys the bronchi. Lesions in the airway in RA may develop as an immune-related process within the bronchial wall and result in hyperplasia of bronchus-associated lymphoid tissue (BALT) (Boyton et al., 2013). In addition to airway lesions, rheumatoid arthritis is associated with various interstitial processes, where UIP is the most frequent phenotype of IP in RA, with nonspecific (NSIP) and desquamative (DIP) interstitial pneumonia and organizing pneumonia (OP), also seen. In RA-UIP, HRCT appearances may demonstrate honeycombing with a peribronchovascular and subpleural distribution. Histopathological analysis has also identified honeycomb-like destructive changes following airway inflammation, in RA-UIP (Sugitani et al., 2018).

In patients with RAIP who have never smoked, emphysema probably develops subsequent to the initiation of destructive interstitial/airway disease and may be related to the high incidence of airway disease in patients with RA. Although the causes underlying the high incidence of airway lesions remain unclear, the “emphysematous” lung lesions noted in rheumatoid arthritis may primarily be related to an airway-centred destructive process.

In assessing the morphogenesis of interstitial pneumonia with emphysema in smokers as well as rheumatoid arthritis with usual interstitial pneumonia in never-smokers, the authors of this paper have presented very important and thought-provoking observations (Jacob et al., 2018). Importtantly, the primary clinical significance of the paper is the demonstration of the poor prognosis associated with emphysema in RAIP even when patients have never smoked.

## Disclosure

The author has received research consultant fee from Boehringer Ingelheim, lecturer fee from Shionogi Pharmaceuticals, and Japanese Ministry of Health, Labor and Welfare Research Fund.
